# Bond Strength of Milled and Printed Zirconia to 10-Methacryloyloxydecyl Dihydrogen Phosphate (10-MDP) Resin Cement as a Function of Ceramic Conditioning, Disinfection and Ageing

**DOI:** 10.3390/ma17092159

**Published:** 2024-05-05

**Authors:** Wolfgang Bömicke, Franz Sebastian Schwindling, Peter Rammelsberg, Stefan Rues

**Affiliations:** 1 Department of Prosthetic Dentistry, University of Heidelberg, 69120 Heidelberg, Germany; peter.rammelsberg@med.uni-heidelberg.de (P.R.); stefan.rues@med.uni-heidelberg.de (S.R.); 2 Department of Prosthetic Dentistry, Medical University Innsbruck, 6020 Innsbruck, Austria; sebastian.schwindling@tirol-kliniken.at

**Keywords:** lithography-based ceramic manufacturing, tribochemical silicatisation, additive manufacturing, adhesive cementation, zirconium dioxide

## Abstract

This study aimed to assess the suitability of printed zirconia (ZrO_2_) for adhesive cementation compared to milled ZrO_2_. Surface conditioning protocols and disinfection effects on bond strength were also investigated. ZrO_2_ discs (n = 14/group) underwent either alumina (Al_2_O_3_) airborne particle abrasion (APA; 50 µm, 0.10 MPa) or tribochemical silicatisation (TSC; 110 µm Al_2_O_3_, 0.28 MPa and 110 µm silica-modified Al_2_O_3_, 0.28 MPa), followed by disinfection (1 min immersion in 70% isopropanol, 15 s water spray, 10 s drying with oil-free air) for half of the discs. A resin cement containing 10-methacryloyloxydecyl dihydrogen phosphate (10-MDP) was used for bonding (for TSC specimens after application of a primer containing silane and 10-MDP). Tensile bond strength was measured after storage for 24 h at 100% relative humidity or after 30 days in water, including 7500 thermocycles. Surface conditioning significantly affected bond strength, with higher values for TSC specimens. Ageing and the interaction of conditioning, disinfection and ageing also impacted bond strength. Disinfection combined with APA mitigated ageing-related bond strength decrease but exacerbated it for TSC specimens. Despite these effects, high bond strengths were maintained even after disinfection and ageing. Adhesive cementation of printed ZrO_2_ restorations exhibited comparable bond strengths to milled ZrO_2_, highlighting its feasibility in clinical applications.

## 1. Introduction

Biological, economic, and esthetic considerations have led to the increasing replacement of traditional metal-ceramics by all-ceramic materials in the fabrication of (in particular fixed) dental prostheses [1,2]. Zirconia (ZrO_2_) is playing a leading role in this trend. The reasons for this include the high load-bearing capacity of the material due to its excellent strength [1], superior marginal adaption [3] and the possibility of monolithic processing in reduced thicknesses, which has been made practical by developments in colouring technique and the availability of pre-coloured blanks. All this has led to an expansion of the range of applications of the material to include the realisation of complex restoration geometries and minimally invasive restorations [4].

For ZrO_2_ single crowns and fixed partial dentures (FPDs), a recent review of the effect of the luting agent used to seat the restorations concluded that high survival rates can be achieved when conventional cements are used for luting [5]. However, the selected studies suggested even greater success with composite or self-adhesive resin cements [5]. In contrast, adhesive cementation is essential for minimally invasive restorations such as vestibular and occlusal veneers (tabletops) and resin-bonded FPDs, as the aim is to achieve a defect-adapted preparation rather than to generate axially parallel surfaces that create a wedging effect during cementation and thus hold the restoration in place. Instead, the long-term retention of these restorations is based on adhesion, making the quality of the adhesive bond a primary prognostic criterion [6].

In the case of ZrO_2_ restorations, there were initial concerns about whether they could be effectively adhesively bonded because the material lacks a glass phase and cannot be etched with hydrofluoric acid, so there was no established method of creating a retentive micro-relief to prepare for a micromechanical bond. Silanisation to create a chemical bond was also ineffective. Today, we have advanced to the point where a permanent bond between a composite cement and ZrO_2_ is possible. The prerequisites are (1) an Al_2_O_3_ airborne particle abrasion process that cleans, roughens and simultaneously enlarges the surface to prepare it for micromechanical bonding and (2) the use of a composite resin or primer containing a functional monomer that enables chemical coupling of the adhesive to the ZrO_2_ substrate [7]. The functional phosphate monomer 10-methacryloyloxydecyl dihydrogen phosphate (10-MDP) has been used successfully in this context [7,8]. As an alternative to a pure Al_2_O_3_ blasting process, tribochemical silicatisation can be used as a surface pretreatment for ZrO_2_ bonding [9]. The combination of tribochemical silicatisation of the ZrO_2_ surface with a silane and 10-MDP-containing primer and 10-MDP-containing cement proved to be effective in achieving a sufficiently strong long-term bond [10]. There is clinical data to support both approaches [4,7,11]. However, Al_2_O_3_ blasting is supported by a larger number of cases and a longer observation period.

More recently, ZrO_2_ has become available as a material for additive manufacturing of dental restorations using the printing process [12,13]. Compared to subtractive milling, ZrO_2_ printing offers material savings/increased cost effectiveness [14] and the ability to create complex geometries with greater accuracy of fit [15] and allows for thinner restorations by eliminating the risk of material damage from the milling process [16]. Overall, these fabrication characteristics support a minimally invasive approach with printed ZrO_2_ restorations but only if a bond strength similar to that of milled material can be achieved. However, while more and more studies are focusing on the mechanical strength [17,18,19,20,21,22], fit [15,23,24,25] and biocompatibility [26,27,28] of the printed material, there has been little research into the adhesion of resins with printed ZrO_2_ [29,30,31]. 

In the meantime, studies have shown very well which cleaning methods work on contaminated ZrO_2_ surfaces [32]. However, little is known about the disinfection of uncontaminated but mechanically conditioned surfaces, although this is inevitable under hygienic conditions, so that disinfection is also of interest as a factor possibly influencing bond strength.

Therefore, the aim of the present study was to determine the influence of the ZrO_2_ type (milled, printed), ceramic conditioning method (Al_2_O_3_ blasting, tribochemical silicatisation), disinfection and artificial ageing on the strength of the ZrO_2_–resin cement bond. The null hypothesis was that none of these variables would affect the resin bond strength to the ZrO_2_.

## 2. Materials and Methods

The materials used in the study and their specifications are listed in Table 1. All materials were used in accordance with the manufacturer’s instructions for use. Test specimen preparation, including all bonding procedures and tensile testing, was performed by one trained person in the position of a physical-technical assistant.

A total of 224 ZrO_2_ discs (diameter 8.4 mm, thickness 3.4 mm) were fabricated, 112 from milled ZrO_2_ (MZ, IPS e. max ZirCAD LT, Ivoclar Vivadent, Schaan, Liechtenstein) and 112 from printed ZrO_2_ (PZ, LithaCon 3Y 230, Lithoz, Vienna, Austria), and ground to a uniform surface finish using 220-grit diamond discs in a semi-automatic grinding and polishing machine (Tegramin-25, Struers, Willich, Germany).

Half of the discs were subjected to airborne particle abrasion (APA) with 50 µm Al_2_O_3_ particles (Alustrahl, Omnident, Rodgau Nieder-Roden, Germany) at 0.10 MPa, while the other half were tribochemically silicatised (TSC) in a two-step blasting process (1. Rocatec Pre: 110 µm Al_2_O_3_ particles, 0.28 MPa, 2. Rocatec Plus: 110 µm silica modified Al_2_O_3_ particles, 0.28 MPa, 3M Oral Care, Seefeld, Germany). All blasting was performed at 10 mm distance from the surface at a 90-degree angle. The discs were blackened in advance with a felt-tip pen to ensure that the surface treatment was complete. Blasting agent residue was removed with a strong stream of oil-free air.

Half of the ZrO_2_ discs were then disinfected (D), while the other half were not disinfected (ND). Disinfection consisted of immersion in 70% isopropanol (Carl Roth, Karlsruhe, Germany) for 1 min, followed by a 15 s water spray and drying with a strong stream of oil-free air for 10 s.

Before adhesive cementation, a primer containing silane and 10-MDP (Clearfil Ceramic Primer Plus, Kuraray Europe, Hattersheim, Germany) was applied to the TSC discs. 

Autopolymerising 10-MDP-based resin cement (Panavia 21, Kuraray Europe) was used to adhesively cement acrylic tubes filled with dual polymerising core build-up resin (Rebilda DC, VOCO, Cuxhaven, Germany) to the ZrO_2_ discs. The tubes were filled just prior to adhesive cementation and the core build-up resin light polymerised from four orthogonal positions from the tube surface (40 s per position) using a 1000 mW/cm² cordless pen-style, LED light polymerisation device (SmartLite Focus LED, Dentsply Sirona, Bensheim, Germany). The bonding area was defined by the 3.3 mm internal diameter of the tubes. Adhesive cementation was performed under a constant load of 7.5 N in a special cementing device that ensured perpendicular alignment of the acrylic tube with the ZrO_2_ disc. After 10 min, the test specimens were transferred to an incubator and stored at 37 °C under 100% humidity for 24 h. 

Half of the specimens were then subjected to a tensile test to determine the bond strength (initial bond strength). The other half of the specimens were artificially aged prior to the bond strength test. The ageing protocol consisted of water storage at a constant temperature of 37 °C interrupted by 7500 thermocycles at 6.5 °C and 60 °C with a dwell time at each temperature of 45 s and a total transfer time of 7.5 s (Thermocycler TC 1, SD Mechatronik, Feldkirchen-Westerham, Germany). The periods of water storage at 37 °C and the period of thermocycling of the test specimens added up to a total of 30 days of storage of the test specimens in water, and the specimens were alternated between the storage conditions according to the following scheme: 4 days of 37 °C water storage, 9 days of thermocycling, 17 days of 37 °C water storage.

The tensile test was performed in a universal testing machine (Z005, Zwick/Roell, Ulm, Germany) at a crosshead speed of 1 mm/min using a moment-free pull-off device. The bond strength (MPa) was calculated by dividing the force (N) applied when the specimen debonded by the bonding area (8.55 mm²). It was defined that test specimens which debonded spontaneously prior to tensile testing (pre-test failures) would be included in the statistics at 0 MPa.

Debonded specimens were optically evaluated with a digital microscope (ZEISS Smartzoom 5, Carl Zeiss, Oberkochen, Germany) at 75× magnification for relative adhesive failure (%) in the bonding area using special measurement software (ZEN core 3.2, Carl Zeiss).

Additional ZrO_2_ discs (not used for bond strength testing) were prepared to evaluate the surface morphology produced by the different ceramic conditioning methods (APA, TSC) qualitatively by scanning electron microscopy (SEM) and quantitatively by surface roughness measurement. SEM (JSM-6510, JEOL, Eching, Germany) was performed with magnifications of 500×, 1000× and 5000× and an acceleration voltage of 5 kV. Average surface roughness R_a_ and ten-point height R_z_ (R_z(iso)_ [33]) were measured using a tactile profilometer (MarSurf GD 140, Mahr, Göttingen, Germany). The roughness of the discs was evaluated along 6 measuring tracks for 2 discs of each surface treatment (including the 220-grit diamond polished baseline surface) and ZrO_2_ type. Each track had a length of 5.6 mm and was measured 3 times. For two perpendicular directions, each 3 tracks were arranged parallel to each other (1 mm distance). Each track was divided into seven intervals and evaluation of roughness parameters took place on a 4 mm long track without the first and last interval. Gauss-filtering took place for wave lengths above λ_c_ = 0.25 mm. 

Bond strength data were verified for normal distribution (Shapiro–Wilk test) and homoscedasticity (Levene test). The influence of ZrO_2_ type, ceramic conditioning method, disinfection, and ageing on bond strength was analysed by multifactorial analysis of variance (ANOVA). A one-way ANOVA with test group as the independent variable, followed by a post hoc Tukey test, was used to pairwise compare test groups for bond strength. For all tests, a *p* < 0.05 was considered statistically significant.

## 3. Results

### 3.1. Bond Strength

**Figure 1:**
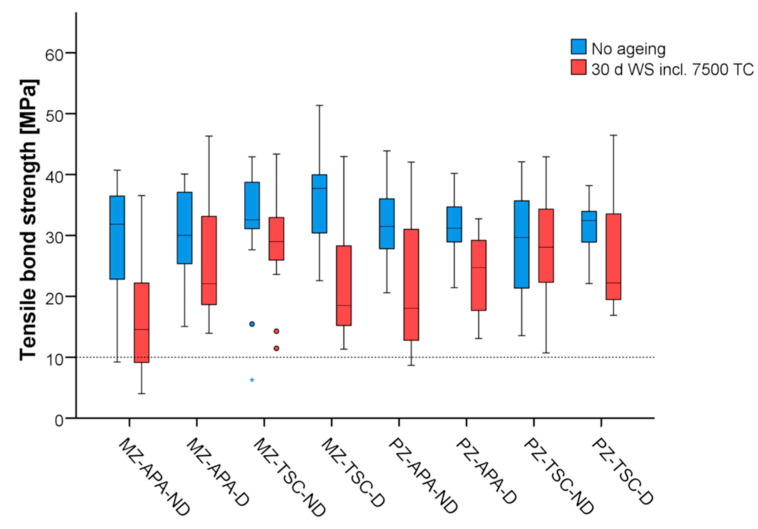
Whisker and box plots of bond strengths in study groups (n = 14 per ageing subgroup). Dotted line marks empirical 10 MPa threshold for clinical recommendation. MZ: milled zirconia, PZ: printed zirconia, APA: airborne particle abrasion, TSC: tribochemical silicatisation, ND: not disinfected, D: disinfected.

All specimens could be tested for tensile bond strength (no pre-test failures occurred). The bond strengths measured in the study groups are shown graphically in Figure 1 and listed in Table 2. 

A statistically significant effect on bond strength was found for ceramic conditioning method (*p* = 0.007) and ageing (*p* < 0.001) as well es for the interaction of ceramic conditioning method, disinfection and ageing (*p* = 0.006) (Table 3).

Mean initial bond strengths ranged from 29.2 MPa to 36.2 MPa for MZ and from 28.7 MPa to 32.1 MPa for PZ. There was no statistically significant difference among the subgroups without ageing (*p* ≥ 0.643). Aged specimens had lower mean bond strengths of 16.6 MPa to 28.8 MPa for MZ and 21.6 MPa to 27.6 MPa for PZ. Among the aged subgroups, a statistically significant lower mean bond strength was found for MZ-APA-ND compared to MZ-TSC-ND specimens (*p* = 0.021). The greatest reduction in bond strength due to ageing was observed in APA-ND and TSC-D specimens. This was found to be statistically significant for MZ (MZ-APA-ND: *p* = 0.014, MZ-TSC-D: *p* = 0.007).

### 3.2. Failure Mode

**Figure 2:**
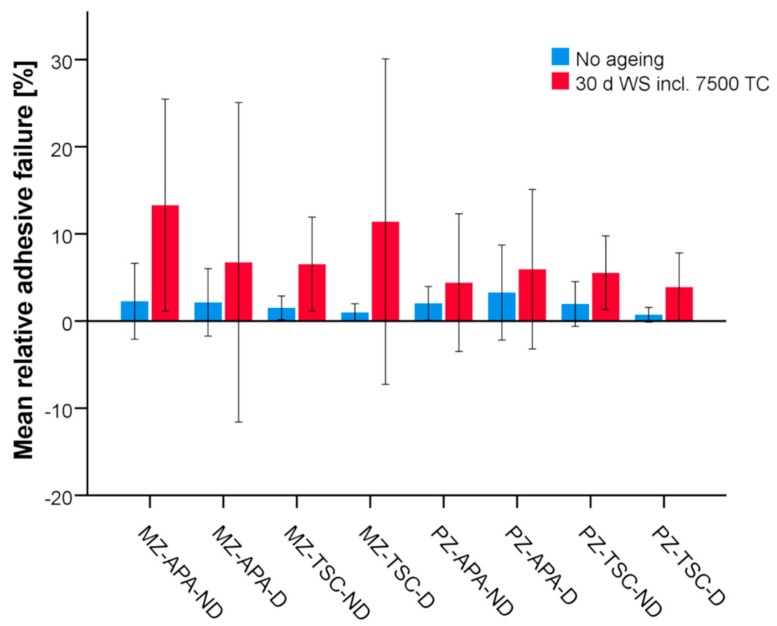
Bar plots of mean relative adhesive failure in study groups (n = 14 per ageing subgroup). Error bars: +/− standard deviation. MZ: milled zirconia, PZ: printed zirconia, APA: airborne particle abrasion, TSC: tribochemical silicatisation, ND: not disinfected, D: disinfected.

The failure mode of the specimens was mainly cohesive in all study groups. The proportion of adhesive failure generally increased with ageing and reached a maximum value of a mean of 13.3% for MZ-APA-ND (Figure 2).

### 3.3. Surface Morphology and Roughness

**Figure 3:**
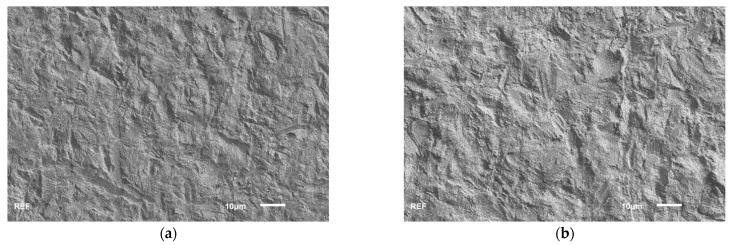

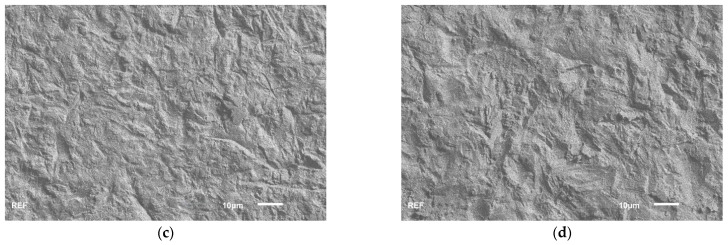
Scanning electron microscopic images of surfaces generated on different zirconia types (milled zirconia, MZ/printed zirconia, PZ) by different conditioning methods (airborne particle abrasion, APA/tribochemical silicatisation, TSC) at 1000× magnification: (**a**) MZ-APA; (**b**) MZ-TSC; (**c**) PZ-APA; (**d**) PZ-TSC.

The SEM analysis showed that the blasting methods used in the study resulted in a comparable surface morphology. The TSC surfaces exhibited a slightly coarser structure compared to the APA surfaces. No differences were observed between milled and printed ZrO_2_ at any chosen magnification. Figure 3 shows the different surfaces at 1000X magnification.

APA approximately doubled the roughness of a 220-grit diamond-polished ZrO_2_ surface and TSC quadrupled it (Table 4). Comparable values were found for milled and printed ZrO_2_ (Table 4).

## 4. Discussion

The objective of this study was to investigate the impact of ZrO_2_ type, ceramic conditioning method, disinfection and ageing on the bond strength between ZrO_2_ and resin. The null hypothesis was that none of these variables would affect the resin bond strength to ZrO_2_. Based on the measured data, the hypothesis was partially rejected. Specifically, there was a statistically significant impact on bond strength for different ceramic conditioning methods, ageing and the combined effect of ceramic conditioning method, disinfection and ageing. Within aged subgroups, a statistically significant difference in mean bond strength was observed, with MZ-APA-ND showing lower bond strength compared to MZ-TSC-ND specimens. Finally ageing led to a statistically significant reduction for MZ-APA-ND and MZ-TSC-D specimens.

In this study, APA and TSC were chosen as the ceramic conditioning methods because they could be considered established for preparing the resin bond to ZrO_2_, not least because they have the most evidence in the literature [34,35]. They also appeared to be particularly suitable because they are easy to apply under practical conditions [10]. A control group without mechanical surface conditioning was not used in this study because it is well known that omitting mechanical roughening of the ceramic substrate results in significantly lower bond strength [35,36]. Recent reviews have shown TSC to outperform APA in achieving stronger bonds with ZrO_2_ [9,34,37,38]. One of these reviews specified, however, that in combination with an MDP resin cement, APA and TSC may be equivalent in terms of achievable bond strength [38]. This well aligns with the present study’s findings. However, it is important to consider the entire bonding process, not just the physical conditioning step, including the adhesive used [35,38]. Using TSC without a proper silane afterward may be ineffective [39]. At the same time, primers/adhesives containing 10-MDP can create strong chemical bonds with ZrO_2_ [37]. Accordingly, a primer containing both a silane and 10-MDP was used in this study on TSC test specimens. In addition, it has already been shown that such a combination of silane and 10-MDP on TSC ZrO_2_ surfaces did result in stable bond strengths [10,40,41]. 

Irrespective of any possible chemical processes, the higher bond strength of the TSC specimens could have simply been related to the surface morphologies produced by the different blasting processes. In terms of SEM morphology, these were comparable for the printed and milled samples and differed only with respect to the ceramic conditioning method chosen, with the TSC samples having a coarser surface. In the roughness evaluation, the values obtained for R_a_ and R_z_ were approximately twice as high for the TSC samples compared to the APA samples. However, there are no studies that could readily substantiate this assumption, as the effects that can be attributed to surface roughness are generally overlaid by other influencing factors, particularly the choice of cement [38]. Studies in which neither increasing Al_2_O_3_ particle size [42] nor increasing air abrasion pressure [10,36] resulted in increased bond strength when used with an MDP-containing cement or primer provide evidence that absolute roughness might be of minor importance. This assumption is also supported by the results of a recently published study, which showed that not R_a_ but the presence (area %) of nanoscale surface irregularities was the most predominant factor for the strength of the resin-zirconia bond [43].

Another difference between the APA and TSC specimens was the use of a primer containing silane and MDP in the TSC specimens. As mentioned above, this is necessary for the chemical coupling of the resin cement to the applied silicate layer, but it also increases the wettability of the surface of the coated ZrO_2_ substrate [44,45], which in turn may have resulted in improved cement flow onto the ZrO_2_ surface and better mechanical interlocking, and thus increased bond strength in the TSC groups. 

The chosen combinations of ceramic conditioning methods (APA or TSC) and 10-MDP-based resin cement, as used in this study, are bonding protocols for which data are available from clinical trials [4,7,11]. Therefore, it was considered particularly useful to validate them for printed ZrO_2_. In the current study, 10 MPa was used as the threshold for clinically acceptable bond strength. It is assumed that bond strength values above this threshold are sufficient to ensure that, for example, a single-retainer resin-bonded fixed partial denture in the anterior region, for which a minimum bonding area of approximately 30 mm² is recommended [46], will not detach under the occlusal forces that may be exerted on it in this position [47]. Overall, it was found that even after ageing, only very few test specimens were below this threshold, and these were almost exclusively APA test specimens of MZ or PZ without disinfection, which in principle qualifies all bonding protocols tested here for clinical use.

Ageing reduced the bond strength of the test specimens; however, a statistically significant reduction was only found for MZ-APA-ND and MZ-TSC-D test specimens. For artificial ageing, a combination of 30 days of water storage including 7500 thermocycles was used, which in accordance with “ISO/TS 4640:2023, Dentistry, Test methods for tensile bond strength to tooth structure” might be considered as medium-term ageing. It should be noted that bond strength tests comparing this ageing protocol with 6 months of water storage or 150 days of water storage including 37500 thermocycles suggest that, with extended ageing, statistically significant effects could also occur in other test groups, which is why conclusive statements on long-term bond strength cannot be made based on this study. On the other hand, with a maximum decrease in bond strength of approximately 37% and 43% (measured for MZ-TSC-D and MZ-APA-ND specimens, respectively), the protocols tested proved to be effective in terms of bond durability [38] and, in view of the clinical threshold defined above, the absolute bond strengths found still contain significant ageing reserves. The ageing resistance of TSC specimens has been attributed to the hydrolytic stability of the siloxane bonds formed between the silanol groups of the silane and the silica layer deposited on the ZrO_2_ surface [48]. However, ageing effects must also be expected for TSC ZrO_2_ [49]. For 10-MDP, it was shown that it bonds directly to ZrO_2_ not only via ionic bonding but also hydrogen bonding [50]. However, it has also been shown that this bond is subject to hydrolytic degradation over longer periods of time [51]. This, in turn, is relativised by the finding that MDP-containing primers, universal adhesives and resin cements create bonds to ZrO_2_ with acceptable strength after long-term aging, regardless of such hydrolytic processes [52].

In addition to the effects of surface conditioning and ageing, the influence of disinfection was of interest. This had derived from the fact that, in general, the bonding surface has the highest surface energy after conditioning and any contamination should be avoided to achieve the best possible bond quality with the subsequently applied adhesives [32]. For TSC samples, it was found that the silane should be applied to the freshly silicatised ZrO_2_ and that cleaning with water (spray or ultrasonic bath) prior to primer application is not advisable [53]. However, as noted in a recent systematic review, ultrasonic cleaning prior to resin cement application is widely used [37]. Similarly, distilled water, alcohol, acetone, ethanol and isopropanol have been used for 1 to 10 min without consideration of the effect of such cleaning steps on adhesion to ZrO_2_, although all were considered beneficial [37]. As the physical conditioning of the ceramic surface often takes place in the dental laboratory, disinfection is essential for further hygienic processing of the restoration. The only way to avoid this is to carry out all ceramic conditioning steps chairside. Technically, this is possible with the appropriate blasting equipment, but the time required, and the increased cleaning requirements of the treatment room associated with the blasting process argue against this procedure. A statistically significant effect of disinfection was found in this study only in the interaction with ageing and conditioning method, which was expressed as a lower sensitivity to ageing of the adhesive bond for APA samples and, in contrast, an increased sensitivity to ageing for TSC samples. The mechanisms underlying this observation cannot be determined at this time. In principle, it is conceivable that disinfection may have interfered with silane coupling, as has already been shown for the effect of water on a silicatised surface [53], and that hydrolytic processes were able to take place to a greater extent, whereas 10-MDP was less affected. The positive effect of disinfection on the APA specimens could be based on a cleaning effect by removing loose abrasive from the surface and increasing the bond quality accordingly.

In the current study, no effect of the ceramic substrate on bond strength was observed. To the best of the authors’ knowledge, only three other studies have evaluated resin bond strength to printed ZrO_2_ [29,30,31]. Control groups from milled ZrO_2_ were used in two of these studies [29,31]. Zandinejad et al. compared milled ZrO_2_ specimens with printed ZrO_2_ specimens of increasing porosity (0%, 20%, 40%) [29]. The ZrO_2_ specimens were bonded after being wet-polished with 600 grit silicon carbide paper and air-abraded with 50 µm Al_2_O_3_ particles at 0.2 MPa. Contrary to the results of the present study, a statistically significant higher bond strength was found for the unaged group of milled ZrO_2_, whereas for aged (5000 thermocycles) specimens, the bond strength of milled and additive manufactured ZrO_2_ with 0% porosity was at the same level. In addition, it was found that increasing porosity was associated with decreasing bond strength for additively manufactured ZrO_2_. With reference to Branco et al. [54], the authors attributed the initially higher bond strength of milled zirconia to a lower wettability of additively manufactured nanostructured ZrO_2_. Overall, there was a high number of pre-test failures and a predominance of adhesive failure in the specimens, which appears to severely limit the ability to draw conclusions in relation to the ZrO_2_ substrate, rather suggesting that an adhesive system was used that appears unsuitable for ZrO_2_ bonding. Zhang et al. compared the initial bond strength of milled and printed ZrO_2_ with or without airborne particle abrasion using 110 µm Al_2_O_3_ particles at 0.2 MPa [31]. Additionally, a group of printed ZrO_2_ with hexagonal surface microstructures was tested [31]. As expected, APA statistically significantly increased the bond strength of milled and printed specimens, with the printed specimens lagging slightly (but statistically significantly) behind the milled specimens. Interestingly, the establishment of hexagonal microstructures had the same effect on bond strength as APA and ensured that there was no statistically significant difference between APA and microstructured printed ZrO_2_. Dai et al. focused exclusively on the possibilities offered by the introduction of microstructures on the surface of printed ZrO_2_ in terms of the bond strength achievable with a resin cement [30]. There was no milled control. Surfaces that were exclusively microstructured (using grooves or a hexagonal grid) did not reach the bond strength that could be achieved as a result of 0.2 MPa 50 µm Al_2_O_3_ APA after aging with 10,000 thermocycles but exceeded it in the case of the grooved surface when it was additionally subjected to an APA process. 

As mentioned above, the study is limited in terms of its predictive value for long-term bond strength. Other limitations include the method of artificial ageing used in this study and the selection of adhesives tested. In terms of simulating the clinical situation, the ageing protocol differed from a clinical application in that, in addition to storage in a humid environment and thermal and mechanical stress on the bond due to thermocycling, direct mechanical forces would act on the bond in the oral cavity, which was not represented in the study and for which anatomical specimens would typically be used. With regard to the adhesive used, the results are only valid for this adhesive. This is a limitation as there are now many adhesives being promoted for bonding ZrO_2_, which vary greatly in composition, mode of application, and sensitivity to ageing.

The results of this study, in conjunction with the last two studies discussed, are very useful in formulating the need for further research. In principle, the adhesive bond to printed ZrO_2_ seems to be subject to the same factors (in terms of the adhesive used, the influence of surface conditioning and ageing) as to milled ZrO_2_. In the future, however, the possibility of microstructuring printed ZrO_2_ surfaces may reduce or eliminate the need for separate surface conditioning. However, this is dependent on finding a microstructure that allows bond strengths comparable to those achieved using the current standard, i.e., APA or TSC. The importance of chemical bonding should also be re-evaluated in the context of microstructured surfaces.

## 5. Conclusions

High bond strengths were achieved with the surface modifications tested, even after disinfection and ageing. While disinfection in combination with Al_2_O_3_ airborne particle abrasion resulted in a lower loss of bond strength due to ageing, it increased the negative ageing effect for the tribochemically silicatised specimens. Adhesive cementation of printed ZrO_2_ resulted in bond strengths that were comparable to those of milled ZrO_2_.

## Figures and Tables

**Table 1:** Specifications of study materials.

Material	Brand	LOT	Composition (as Disclosed by Manufacturer)	Material
Milled zirconia	IPS e.max ZirCAD LT	X54 580	88–95.5 wt% zirconium oxide (ZrO_2_), 4.5–≤6 wt% yttrium oxide (Y_2_O_3_), ≤5 wt% hafnium oxide (HfO_2_), ≤1 wt% aluminium oxide (Al_2_O_3_), ≤1 wt% other oxides for coloring	Ivoclar Vivadent, Schaan, Liechtenstein
Printed zirconia	LithaCon 3Y 230	N.a.	3 mol% yttria stabilized tetragonal zirconia polycrystal	Lithoz, Vienna, Austria
Multifunctional primer	Clearfil Ceramic Primer Plus	AX0039, 2A0061	>80% ethanol, 3-trimethoxysilylpropyl methacrylate, 10-methacryloyloxydecyl dihydrogen phosphate	Kuraray Europe, Hattersheim, Germany
Adhesive resin cement	Panavia 21	2G0018, 49A0019, 5E0015	Catalyst paste: 10-methacryloyloxydecyl dihydrogen phosphate, hydrophobic aromatic dimethacrylate, hydrophobic aliphatic dimethacrylate, silanated silica filler, colloidal silica, catalysts; Universal paste: hydrophobic aromatic dimethacrylate, hydrophobic aliphatic dimethacrylate, hydrophilic aliphatic dimethacrylate, silanated titanium oxide, silanated barium glass filler, catalysts, accelerators, pigments	Kuraray Europe, Hattersheim, Germany
Oxygen- inhibiting gel	Oxyguard II	480101, 720103, A40106	50–70% glycerol, polyethyleneglycol, catalysts, accelerators, dyes	Kuraray Europe, Hattersheim, Germany
Core build-up resin	Rebilda DC	2133130, 2205471	Catalyst paste: 10–25% urethane dimethacrylate (UDMA), 5–10% 1,12-dodecane dimethacrylate (DDDMA), 2.5–5% bisphenol A-glycidyl methacrylate (BIS GMA), ≤2.5% benzoyl peroxide; Base paste: 10–25% UDMA, 5–10% DDDMA, 2.5–5% BIS GMA	VOCO, Cuxhaven, Germany

**Table 2:** Tensile bond strength [MPa] in study groups.

Study Groups (n = 14 per Ageing Subgroup)	Ageing													
	No Ageing							30 d Water Storage Incl. 7500 TC						
	Mean	SD	Min	Max	Median	25th Pct	75th Pct	Mean	SD	Min	Max	Median	25th Pct	75th Pct
MZ-APA-ND	29.2 ^ab^	9.3	9.2	40.7	31.9	22.8	36.5	16.6 ^c^	10.3	4.0	36.6	14.6	9.2	22.2
MZ-APA-D	29.7 ^ab^	7.4	15.1	40.1	30.0	25.4	37.1	25.2 ^abc^	9.7	13.9	46.3	22.1	18.7	33.1
MZ-TSC-ND	31.7 ^ab^	10.0	6.3	42.9	32.6	31.1	38.7	28.8 ^ab^	8.9	11.5	43.3	29.0	26.0	33.0
MZ-TSC-D	36.2 ^a^	7.5	22.6	51.4	37.7	30.4	40.0	22.9 ^bc^	10.6	11.3	43.0	18.5	15.2	28.3
PZ-APA-ND	32.1 ^ab^	6.8	20.6	43.9	31.5	27.8	36.0	21.6 ^bc^	11.1	8.7	42.1	18.1	12.8	31.0
PZ-APA-D	31.2 ^ab^	5.2	21.4	40.2	31.2	28.9	34.7	24.0 ^bc^	6.8	13.1	32.7	24.7	17.7	29.2
PZ-TSC-ND	28.7 ^ab^	9.6	13.6	42.1	29.7	21.4	35.7	27.6 ^abc^	8.5	10.7	42.9	28.1	22.3	34.3
PZ-TSC-D	32.0 ^ab^	4.5	22.1	38.2	32.5	28.9	34.0	26.8 ^abc^	9.20	16.9	46.5	22.2	19.5	33.5

Different lowercase letters indicate a statistically significant difference in bond strength in the Tukey post hoc test. SD: standard deviation, Pct: percentile, MZ: milled zirconia, PZ: printed zirconia, APA: airborne particle abrasion, TSC: tribochemical silicatisation, ND: not disinfected, D: disinfected.

**Table 3:** Multifactorial analysis of variance for effect of zirconia type, ceramic conditioning method, disinfection and ageing on bond strength.

Source	Typ III Sum of Squares	df	Mean Square	F	*p*
Corrected model	4904.915 ^a^	15	326.994	4.359	<0.001
Intercept	172,656.653	1	172,656.653	2301.463	<0.001
Zirconia type	12.946	1	12.946	0.173	0.678
Ceramic conditioning method	549.472	1	549.472	7.324	0.007
Disinfection	118.306	1	118.306	1.577	0.211
Ageing	2859.215	1	2859.215	38.112	<0.001
Zirconia type * ceramic conditioning method	140.891	1	140.891	1.878	0.172
Zirconia type * disinfection	11.653	1	11.653	0.155	0.694
Zirconia type * ageing	76.928	1	76.928	1.025	0.312
Ceramic conditioning method * disinfection	80.197	1	80.197	1.069	0.302
Ceramic conditioning method * ageing	134.556	1	134.556	1.794	0.182
Disinfection * ageing	7.011	1	7.011	0.093	0.760
Zirconia type * ceramic conditioning method * disinfection	116.540	1	116.540	1.553	0.214
Zirconia type * ceramic conditioning method * ageing	96.534	1	96.534	1.287	0.258
Zirconia type * disinfection * ageing	1.948	1	1.948	0.026	0.872
Ceramic conditioning method * disinfection * ageing	588.806	1	588.806	7.849	0.006
Zirconia type * ceramic conditioning method * disinfection * ageing	109.914	1	109.914	1.465	0.227
Error	15,604.245	208	75.020		
Total	193,165.813	224			
Corrected total	20,509.159	223			

^a^ R-Squared = 0.239 (Adjusted R-Squared = 0.184).

**Table 4:** Mean values [µm] of zirconia surface roughness measurements for different ceramic conditioning methods.

Ceramic Surface Conditioning Method	MZ		PZ	
	R_a_	R_z_	R_a_	R_z_
220-grit diamond disc polishing (starting surface)	0.4763	3.0689	0.4420	2.9333
APA	0.9348	6.2130	0.8377	5.4151
TSC	1.9214	11.0322	1.8998	11.0238

MZ: milled zirconia, PZ: printed zirconia, APA: airborne particle abrasion, TSC: tribochemical silicatization.

## Data Availability

The data on which the results of this study are based can be made available upon reasoned request to the corresponding author.
